# Rigor of Neurovascular Coupling (NVC) Assessment in Newborns Using Different Amplitude EEG Algorithms

**DOI:** 10.1038/s41598-020-66227-y

**Published:** 2020-06-08

**Authors:** Yudhajit Das, Hanli Liu, Fenghua Tian, Srinivas Kota, Rong Zhang, Lina F. Chalak

**Affiliations:** 1 Department of Bioengineering, University of Texas Southwestern Medical Center, Dallas, TX USA; 2 Department of Neurological Surgery, University of Texas Southwestern Medical Center, Dallas, TX USA; 3 Department of Internal Medicine, University of Texas Southwestern Medical Center, Dallas, TX USA; 4 Department of Pediatrics, University of Texas Southwestern Medical Center, Dallas, TX USA

**Keywords:** Electrophysiology, Neurology

## Abstract

Birth asphyxia constitutes a major global public health burden for millions of infants with a critical need for real time physiological biomarkers. This proof of concept study targets the translational rigor of such biomarkers and aims to examine whether the variability in the amplitude-integrated EEG (aEEG) outputs impact the determination of neurovascular coupling (NVC) in newborns with encephalopathy. A convenience sample with neonatal asphyxia were monitored for twenty hours in the first day of life with EEG and near infrared spectroscopy (NIRS)-based cerebral tissue oxygen saturation (SctO2). NVC between aEEG and NIRS-SctO2 was assessed using wavelet transform coherence (WTC) analysis, specifically by the wavelet total pixel number of significant coherences within 95% confidence interval. The raw EEG was converted to aEEG using three different methods: Method (M1) derives from the algorithm by Zhang and Ding. Method (M2) uses a Neonatal EEG Analysis Toolbox (WU-NEAT). Method (M3) extracts output directly from a commercial platform with an undisclosed algorithm. Our results demonstrate excellent agreement with Bland Altman comparisons for WTC-based NVC irrespective of the algorithms used, despite significant heterogeneities in the aEEG tracings produced by three algorithms. Our findings confirm the robustness of NVC wavelet analysis in Neonatal Encephalopathy related to HIE.

## Introduction

The amplitude-integrated EEG (aEEG) has become a very useful clinical device in newborns; new systems are continuously enriching the market with variable algorithms not always disclosed by free access to the public. While the visual pattern recognition of aEEG provides a validated gold standard tool for the clinical use in sick newborns^1,2^, there are no studies to date testing the effect of using different algorithms on the pattern recognition and its effect on clinical practice. For research purposes, similarly, there is no specific standardized algorithm method to quantify the aEEG signal from raw EEG data.

The lack of transparency and multitude of algorithms generating aEEG signals can represent a significant challenge for the rigor of the clinical analysis that relies on aEEG processing, affecting both the clinical pattern recognition^3,4^ as well as research computations of neurovascular coupling based on upper and lower margins of aEEG voltage^4,5^. We have recently reported a novel quantification of neurovascular coupling (NVC) using a wavelet transform coherence (WTC) analysis on the dynamic signals of aEEG and cerebral tissue oxygen saturation (SctO2) in neonatal encephalopathy^4^ where time series from a selected pair of electrodes was directly processed as output from a commercial device.

The current proof of concept investigation aims to further examine the rigor and consistency of the novel WTC analysis for NVC quantification by evaluating the effects of using three different aEEG algorithms to process the raw EEG recordings obtained from infants with neonatal encephalopathy. We compare the variability observed with the three algorithms with respect to (1) aEEG tracings with upper and lower margin amplitudes which affect the clinical background pattern, and (2) research-based, WTC-derived metrics of neurovascular coupling NVC.

## Materials and methods

### Subjects and measurement protocol

To test the effect of the different data-processing algorithms, we selected a convenience sample of eight newborns with hypoxic ischemic encephalopathy (HIE) who had a minimum of twenty hours of EEG monitoring as a standard of care protocol. Infants had a birthweight of ≥1800 g, were ≥36 weeks of gestation, and admitted to the neonatal intensive care unit at Parkland Hospital, Dallas, TX with evidence of fetal acidosis and encephalopathy. Infants had normal MRI outcome and none of these infants had seizures during their recording period. The aEEG and regional cerebral tissue oxygen saturation (SctO2) by near infrared spectroscopy (NIRS-SctO2) of all neonates were simultaneously recorded during the entire course of 20-hour monitoring, which made it feasible to perform dynamic wavelet coherence analysis. Eight EEG electrodes were placed on the newborns’ scalps at C3, C4, P3, P4, O1, O2, Cz, and Fz, according to the 10–20 international system. EEG signals from all 8 electrodes were recorded at a sampling rate of 256 Hz and then amplified and filtered within a frequency band of 0.1-100 Hz. For this specific study, time series from a cross-cerebral electrode pair (C3-C4 in the central region) were used for our data analysis for all the neonates. Both EEG and NIRS-SctO2 signals were interfaced with a multi-device synchronization platform (Moberg Research, Inc., PA, USA) for simultaneous recording of two modalities and then saved for off-line analysis using MATLAB (Mathworks, Inc., MA, USA). The study was approved by the Institutional Review Board of the University of Texas Southwestern Medical Center and informed consent was obtained from parents of each newborn before enrollment.

### Data preprocessing for three methods to acquire aEEG

**Figure 1:**
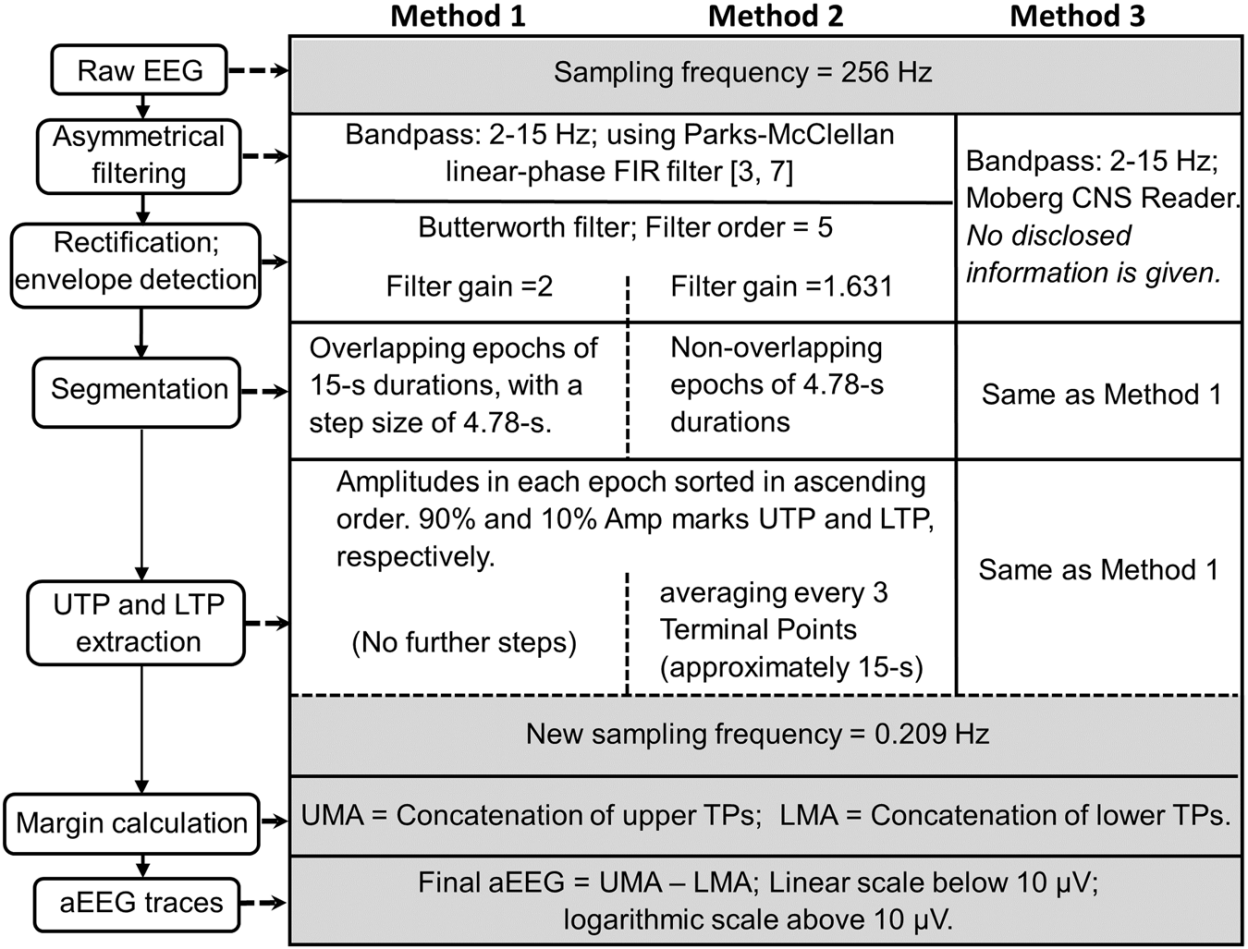
Overview of different steps among three algorithms, showing how to obtain the same sampling rate for aEEG and NIRS. TPs: terminal positions; UTP: upper terminal positions; LTP: lower terminal positions; UMA: upper margin amplitudes; LMA: lower margin amplitudes.

As mentioned earlier, recent commercial EEG systems from different medical device manufacturers have often embedded aEEG modules. While they continuously provide clinicians with advanced and convenient tools, each has their own undisclosed algorithms to obtain aEEG for targeting on specific medical applications of neonates. In general, these methods or algorithms share common or similar steps to preprocess raw EEG data before making the conversion to aEEG which are transparent and briefly summarized below, with a schematic comparison shown in Fig. 1. A finite impulse response (FIR) band-pass filter was used to eliminate low-frequency physiological artifact and to attenuate high-frequency signals over 15 Hz that can arise from muscle activity, power line interference, and other unwanted noises. This filter was digitally implemented to mimic the analog filters of Cerebral Function Monitor (CFM), slightly attenuating the dominant delta-wave patterns^3^. The filtered signal was next passed through a peak sensitive rectifier for peak-to-peak rectification.

After these common steps, different peak detection and data smoothing algorithms for quantifying aEEG are used depending on the device and manufacturer, which are not standardized and often not even disclosed^3,6^. To examine the effect of different aEEG tracing algorithms on the NVC analysis in this study, we used two published algorithms and compared them with an additional third commercial platform/device (Moberg) algorithm. Specifically, the three methods used are summarized below:

### Method 1 (M1)

This method was based on the algorithm reported by Zhang and Ding, with a step-by-step signal-processing approach to calculate aEEG upper and lower margins from raw EEG data^3^.

1. ***Step 1*** – Asymmetrical filtering: An asymmetrical, flat band-pass, linear phase FIR filter was designed using Parks-McClellan algorithm^7^ to compensate the attenuated energy of nonrhythmic components at each frequency^3^. This filter enabled us to perform weighted amplification to EEG signals in the frequency range of 2 - 15 Hz, while simultaneously attenuating low-frequency artifacts.
2. ***Step 2*** – Rectification and Envelope detection: The asymmetrically filtered data were then passed through a peak-sensitive rectifier to undergo full wave rectification for evaluation of the absolute value/amplitude. Since the boundary or envelope of the EEG waveform is the key to characterize the tendency of amplitude changes, envelope detection was implemented on the rectified signal using a 5^th^ order Butterworth filter. This process enabled to extract a smooth curve approximately drawn through the peaks of the rectified EEG tracings and concisely outlined the amplitude feature of the raw EEG signals. A gain factor of 2 was then applied on the envelope when obtaining 256 Hz aEEG signal.
3. ***Step 3*** – Segmentation: This step was designed to achieve a bird’s-eye overview of the long-term aEEG tracing. This part of processing in our method was slightly modified from the original paper to achieve a target sampling frequency of 0.209 Hz, the frequency used in acquiring SctO2 signal from the newborns, in order to perform NVC between them. Thus, instead of taking a non-overlapping sliding window of 15 s^3^, we segmented the envelope of the rectified EEG into overlapping epochs of 15-s durations with a step size of 4.78 s in order to compress the envelope tracing in time (time-scale ref. ^6^ cm/h).
4. ***Step 4*** – Terminal points extraction: The amplitudes in each epoch were thereafter sorted in ascending order of magnitude. The maximum and minimum amplitudes in each epoch should ideally be chosen as upper and lower terminal points, (UTP and LTP), respectively. However, in order to ensure robustness of the algorithm against high and low amplitude noises, the 90^th^ and 10^th^ percentile of the sorted data were defined as UTP and LTP, respectively, of the associated aEEG tracings, for each 15-s sliding, rectangular time window. Choosing an overlap time of 10.22-s for the sliding window, we obtained maximum and minimum peak extractions in each 4.78-s duration. A vertical line was made for each pair of UTP and LTP to represent a 4.78-s epoch. In this way, we were able to achieve a sampling rate of 0.209 Hz (i.e., 1 data point per 4.78-s), which matched the acquisition rate of SctO2 signal, for performing WTC.
5. ***Step 5*** – Margin calculation: Then, a curve was drawn through the peaks and troughs, respectively, to obtain smooth upper and lower margin amplitudes (UMA and LMA, respectively) of aEEG.
6. ***Step 6*** – aEEG traces: The processed aEEG tracings were plotted using a semi log-scale (i.e., base 10) for y-values larger than 10 µV to reduce the dynamical range of large fluctuations of raw EEG, while keeping a linear scale for y values between 0-10 µV to accurately depict the low amplitudes. By the end, the time-varying tracings of differences between UMA and LMA were used for aEEG values to quantify WTC and thus NVC.

### Method 2 (M2)

This method made use of Washington University-Neonatal EEG Analysis Toolbox (WU-NEAT). WU-NEAT is an open source, MATLAB-compatible, clinically validated toolbox; it is encapsulated in an easy-to-use graphical user interface (GUI) to quantify both aEEG and spectral edge frequency for assisting collaborative research in neonatal EEG with limited channels^6^. We utilized WU-NEAT as a second method/algorithm, M2, to calculate aEEG from our raw EEG data. The processing steps were similar to those in M1 and are briefly summarized as three steps, as follows:

1. ***Step 1*** – Asymmetric filtering***:*** Same as M1, raw EEG data were first passed through an asymmetric band-pass filter (Parks-McClellan linear-phase FIR filter), strongly attenuating the signal below 2 Hz for low-frequency physiological artifact, such as respiratory and heart artifact, and above 15 Hz to remove muscle activity and power line noise. The band-passed signals within 2–15 Hz underwent gradual amplification with a slope of 12 dB/decade to compensate for the diminished amplitude due to the scalp and skull attenuations.
2. ***Step 2*** – Rectification and envelope detection***:*** Similar to Step 2 of M1, the band-passed EEG signals were rectified, followed by envelope extraction using a 5^th^ order low-pass Butterworth filter with zero-phase filtering. The resulting aEEG tracings were obtained by applying a gain of 1.631 on the envelope.
3. ***Step 3*** – Segmentation: This step was designed in order to achieve a bird’s-eye overview of the processed aEEG signals (with a 256-Hz sampling rate) after Step 2. The original method in WU-NEAT recorded upper and lower terminal points in every 3.12 s, followed by a simple moving average over 5 such adjacent terminal points to form a smoothed 15.6-s epoch (3.12 s × 5 = 15.60 s). Specifically, for our study, we modified this down sampling technique by segmenting each 256-Hz, 15-s EEG data set into three of 4.78-s epochs to achieve the needed sampling rate of 0.209 Hz.
4. ***Step 4*** – Terminal points extraction: The amplitudes in each 4.78-s epoch, which contained 1224 adjacent EEG data points (=4.78 s × 256 Hz ~ 1224) were sorted in their ascending order of magnitude. The amplitudes of the 90^th^ and 10^th^ percentile of the sorted data were defined as the UTP and LTP to represent each 4.78-s epoch. Next, a simple overlapping moving average with a step of 4.78-s was performed over every 3 upper and lower adjacent terminal points (covering approximately 15-s epoch) to obtain smoothed aEEG tracings.
5. ***Step 5*** – Margin calculation: Same as M1, upper and lower representative aEEG margins were obtained by temporal concatenation of all UTP and LTP respectively.
6. ***Step 6*** – aEEG tracing: Same as M1, a linear scale was used from 0–10 µV and a semi-log scale used from 10–100 µV, following the conventional aEEG presentation format. The time-varying tracings of the differences between UMA and LMA were used for aEEG values to quantify WTC and thus NVC.

### Method 3 (M3)

Direct aEEG output tracings were attained from a commercial synchronization platform with undisclosed algorithm (CNS Reader, Moberg ICU Solutions, Ambler, PA). Moberg ICU Solutions is a medical device manufacturing company that specializes in neuromonitoring and patient information management. The Component Neuromonitoring System (CNS Monitor) is commercially available to display multiple clinical/vital parameters that are collected simultaneously from various medical devices. CNS Reader is a Windows-based software application designed as a companion to the CNS Monitor that facilitates data collection, data recording, and data analysis for multimodal devices, with a focus on EEG.

***Step 1 and 2*** – Pre-processing and Conversion of 256-Hz EEG data into aEEG with the same frequency**:** CNS Reader has inbuilt functions that automatically perform band-pass filtering (2–15 Hz) followed by line noise removal, rectification, and detection of the amplitude margins on raw EEG data. The output parameters of CNS-processed aEEG can be downloaded as a data file for further off-line analysis in MATLAB. The process or algorithm of conversion from raw EEG into aEEG (Step 1 and 2) has not been disclosed by the company. The 256-Hz aEEG tracings directly outputted from the CNS Monitor/Reader were downloaded for our next-step analysis.

As shown in Fig. 1, all the processing steps (i.e., ***Step 3*** to ***Step 6***) in M3 were the same as M1.

**Figure 2:**
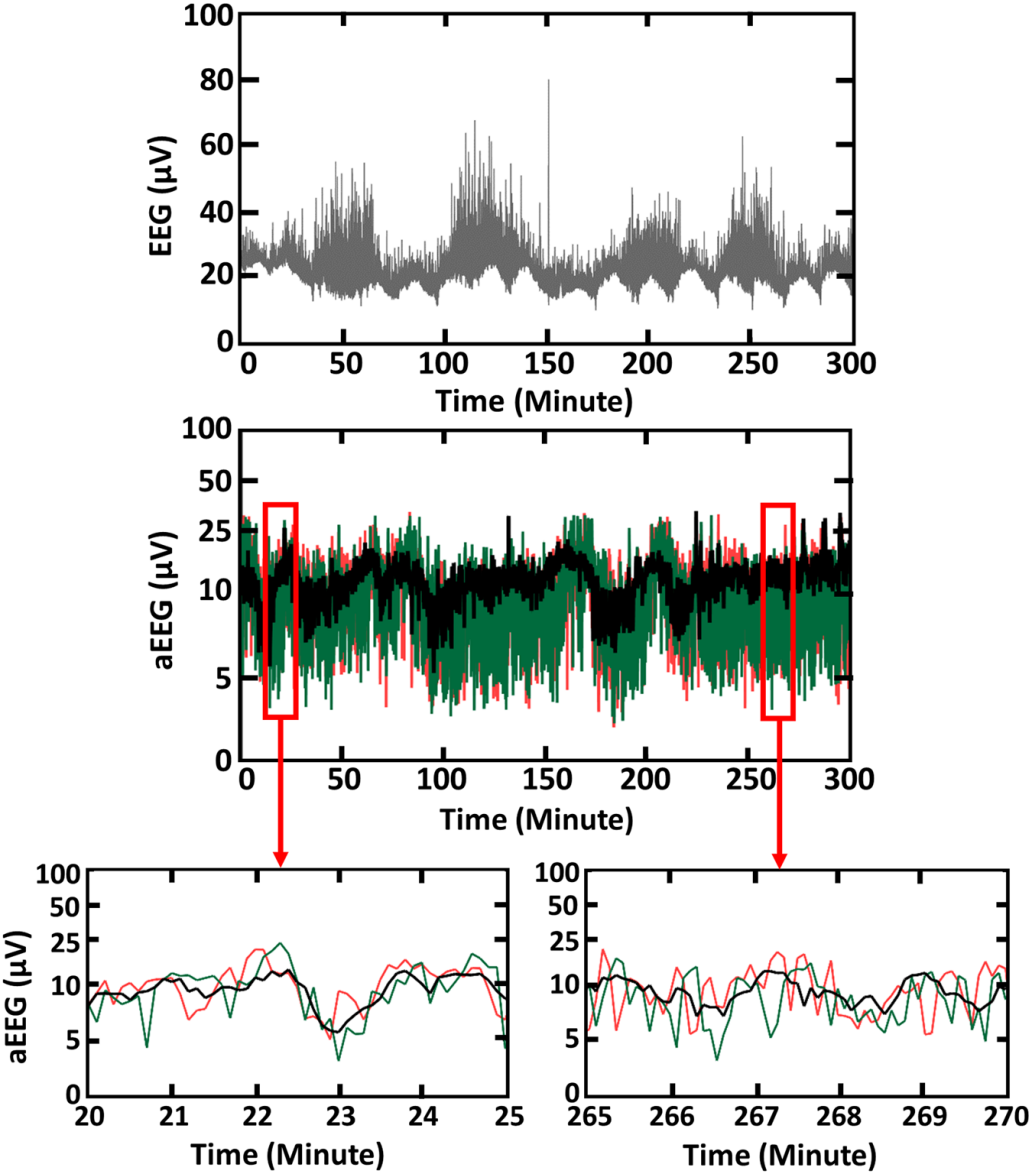
Top illustrates an example of 5-hour raw EEG tracings recorded with a 256-Hz sampling frequency. Middle shows 3 aEEG tracings obtained using M1 (red), M2 (black), and M3 (green). Last shows two highlighted sections of 5-minute each, segmented from the same aEEG tracing derived from M1(red), M2 (black), and M3 (green), respectively.

As an example, Fig. 2 top shows a trace of 5-hour raw EEG signal, sampled at 256-Hz from a normal reference control neonate. The y-axis of this figure represents amplitude of pre-processed EEG in micro-volt and is plotted with a linear scale, while x-axis represents time in minute. Figure 2 middle shows three aEEG tracings derived from the EEG trace shown above using M1 (red), M2 (black), and M3 (green), respectively. To clearly examine the differences among three aEEG curves, we extracted two-time segments with a period of 5-minutes for each. This example is consistent with previous reports ^5^and illustrates how even though all three aEEG were derived from the same EEG tracing, the amplitudes and/or envelop values of the aEEG vary amongst the three methods.

### Quantification of NVC using the wavelet transform coherence (WTC) statistical methods

To determine whether variations in aEEG tracings affect the NVC between aEEG and SctO2, we used WTC analysis between the spontaneous oscillations of aEEG and NIRS-SctO2 as we recently published in Neonatal Encephalopathy (NE)^4^. A 95% confidence interval was chosen to identify regions of interest (ROIs) within the time-scale map. These ROIs mark or label statistically significant coherence reflecting NVC across different times within an upper to lower scale range for each of the three SctO2→aEEGs pairs. Specifically, we used a MATLAB-based software package^8^ to perform WTC analysis between the spontaneous oscillations of NIRS-SctO2 and aEEG (SctO2 → aEEG), each aEEG derived from the same raw EEG time series using each of M1, M2 and M3, respectively, marked as SctO2 → aEEG_M1_, SctO2 → aEEG_M2_, and SctO2 → aEEG_M3_. Using a time-scale (equivalent to time-frequency) domain, NVC was assessed by wavelet metric estimation of total pixel number of significant coherence.

Further, to quantify NVC results derived from three different aEEG methods, we introduced, implemented, and assessed a WTC-derived index in the time-scale domain, *Pix*. Specifically, we denoted the pixel number of significant coherences across all scales. All pixels that had values of squared cross-wavelet coherence R^2^ significantly higher than the simulated background noise (p < 0.05) were identified and marked as statistically significant coherence contours^8^. Then, we quantified total pixels (*Pix*_*total*_) by summing respective pixel numbers across all wavelet time scales within the entire WTC time-scale domain (outside of the cone of inference, COI)^8^. Finally, a coefficient of variation (*COV*) was introduced to evaluate levels of variation and/or effect of different aEEG algorithms on WTC-based NVC.

*COV* was defined as $COV=\frac{SD}{mean\,of\,significant\,coherence\,Pixels\,}$, where *SD* stands for standard deviation of significant coherence pixels across entire scale from the mean values of significant pixels averaged over those determined with the three algorithms.

Statistical analysis to compare agreement between the methods was performed using Bland Altman curves to detect differences larger than one standard deviation from the mean.

## Results

### Effect of the processing algorithm on the clinical pattern of the aEEG

**Figure 3:**
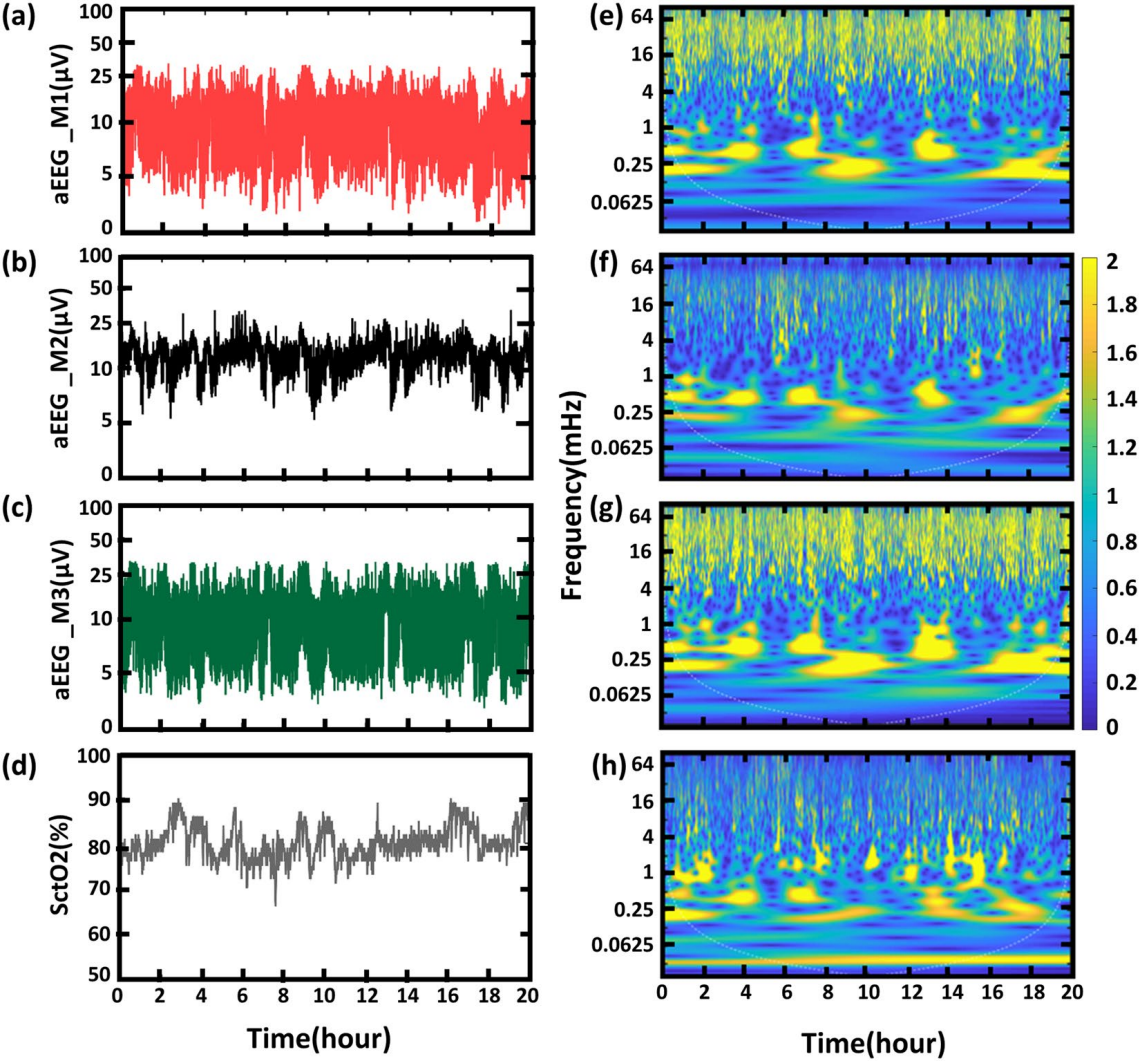
An example of three aEEG tracings in μV obtained using (**a**) M1, (**b**) M2, and (**c**) M3 of a neonate (neonate #1) with normal MRI outcome. (**d**) SctO2 signal recorded simultaneously. (**e**–**h**) Wavelet transformation of the corresponding aEEG tracings derived using (**e**) M1, (**f**) M2, (**g**) M3 and (**h**) SctO2, respectively, where y-axis represents Frequency in mHz, and the color represents the amplitude of power after CWT.

We quantified three aEEG tracings in a convenience sample of eight newborns with HIE across 20 hours of monitoring period in the first day of life using the three aEEG algorithms. As an example, Fig. 3 shows a comparison of the time and time-frequency domain quantifications from neonate #N1 of different aEEG traces along with SctO2 signal recorded simultaneously with the EEG signal. Figure 3 highlights a large variability from using each of M1, M2 and M3 in both lower and upper amplitude margins of these tracings. Specifically, aEEG curves obtained in the same patient using M2 produced a narrow bandwidth and a normal continuous pattern, while M1 or M3 resulted in a discontinuous tracing. The example highlights how the discrepancies in algorithm can result in a different aEEG pattern in the same patient, each being associated with a different therapy and prognosis implication.

### Effect of the processing algorithm on research WTC-derived NVC

Next, we performed time-frequency analysis of the three aEEG tracings derived from M1, M2 and M3, as well as their respective SctO2 signal using continuous wavelet transform (CWT) and analytic Morlet wavelet^3,8^. The corresponding time-frequency spectrograms are shown in Fig. 3(e–h), where the x-axis represents time in hour, the y-axis represents frequency in mHz, and the color scale represents the amplitude of power after CWT. Despite the variability in aEEG tracings in Fig. 3(a–c), consistent patterns are demonstrated in the frequency range of 0.1 mHz to 2 mHz with the spectrograms obtained from the three algorithms marked by dashed lines in Fig. 3(e–g), as well as for the SctO2 spectrogram Fig. 3(h).

**Figure 4:**
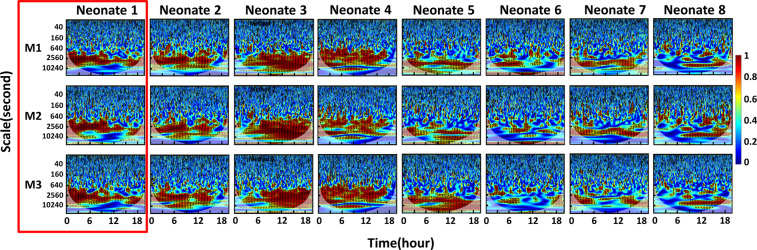
Time-scale coherence maps of NVC in the selected cohort. The x-axis represents time in hours, the y-axis represents scale in seconds, and the color scale represents the amplitude of WTC coherence, R^2^. The areas with significant NVC (p < 0.05) are contoured with black lines and filled by red color within the time-scale WTC maps. WTC-based SctO2-aEEG coherence (*R*^2^) are plotted for each one of the 8 neonates (Neonate #: N1-N8). Each vertical column represents the results derived from three aEEG methods, respectively, highlighting the similarity in NVC pattern for each newborn among *R*_SctO2→aEEG_M1_, *R*_SctO2→aEEG_M2_, and *R*_SctO2→aEEG_M3_.

Furthermore, the WTC-based time-scale coherence maps/spectrograms obtained from the same above patient highlighted various aEEG pattern interpretation (#N1) are illustrated in Fig. 4 red box, comparing effects of three aEEG processing methods on NVC. The figure highlights the consistent time-scale patterns between aEEG and SctO2 represented in the scale range of 640-10240 s (0.1–1.6 mHz).

At the group level, Fig. 4 shows the corresponding time-scale coherence of NVC, R^2^, for all eight neonates highlighting a full range of NVC. Notably, neonates N1-N4 have high NVC coherence while neonates N5-N8 have lower NVC. Despite the large physiological NVC variability observed within multiple patients, consistent coherence patterns were observed specifically in the scale range of 640-10240-s for each neonate depicted, regardless of the aEEG processing methods used.

### Calculation of the total number of pixels within statistically significant contours across entire scale

**Figure 5:**
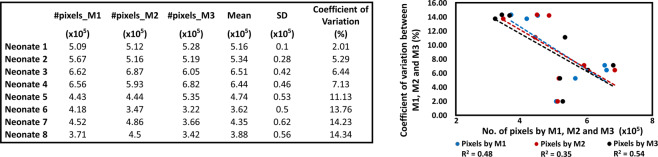
Left- Total number of significant coherence pixels derived with M1, M2 and M3 across the entire scale (*Pix*_*total*_) for each of eight neonates. Right- Relationships of total number of significant pixels (*Pix*_*total*_) derived by M1 (blue), M2 (red) and M3 (black) vs respective *COV*s. Dashed lines represent color-matched regression lines for each case.

Figure 5 tabulates the total number of pixels that are significantly coherent between SctO2 and aEEG identified by three methods across entire scale (*Pix*_*total*_) and the corresponding *COV* values between the methods ranging from 2.01% (Neonate 1) to 14.34% (Neonate 8) for the entire scale. This highlights that high significant coherence with more pixels (such as in N1-N4) is associated with a smaller *COV* value. Figure 5 plots the total number of significant pixels, *Pix*_*total*_, identified by M1, M2 and M3, versus respective *COV* values for all 8 neonates, demonstrating a negative linear relationship between them. Figure 5 indicates that variations in WTC-derived pixels count caused by different aEEG algorithms are associated with the degree of NVC between aEEG and SctO2. Stronger NVC is associated with the least variation.

**Figure 6:**
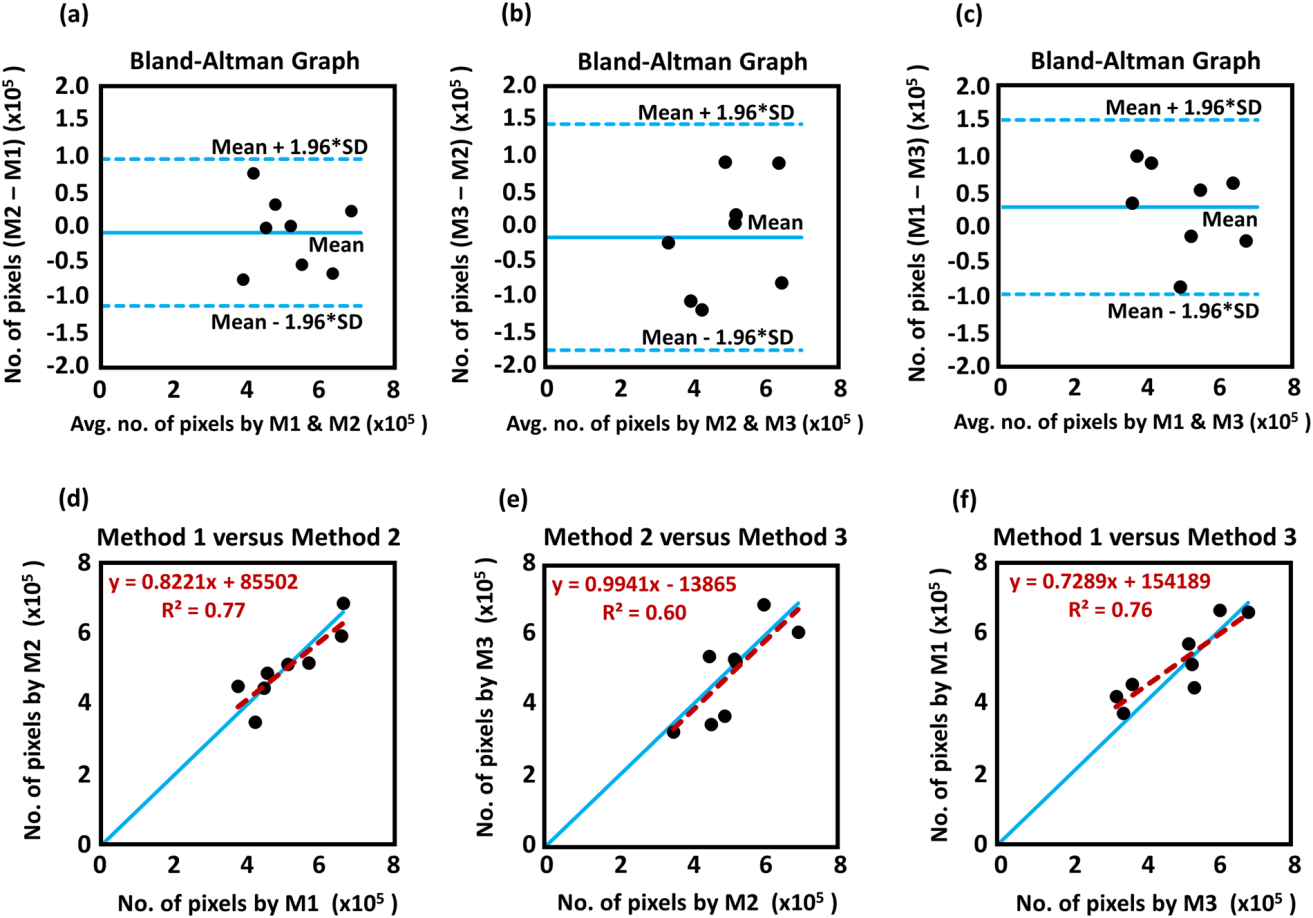
Comparison of WTC results based on aEEG values derived from M1, M2, and M3 using the entire range of wavelet scale. (**a**–**c**) Bland-Altman plots to compare WTC agreements between paired aEEG methods: (**a**) M1 vs. M2, (**b**) M2 vs. M3, and (**c**) M1 vs. M3. The horizontal upper and lower blue lines mark the limits of agreement, defined as the mean difference ± 1.96 SD. (**d**–**f**) A comparison of total numbers of significant coherence pixels derived from paired aEEG methods: (**a**) M1 vs. M2, (**b**) M2 vs. M3, and (**c**) M1 vs. M3. A blue “line of identity” is given in each panel as perfect match between the results from the two respective methods. The red line in each panel shows the linear regression.

We utilized Bland-Altman plots to compare the total number of significant coherence pixels (#_pixels) over the entire wavelet scale range by M1, M2 and M3, as shown in Fig. 6(a–c). Results indicate that the differences in #_pixels are within the one standard deviation limit for all neonates regardless of the pair of methodology M1 vs. M2, M1 vs. M3, or M2 vs. M3. This observation implies that regardless of differences in aEEG processing algorithms, a significant level of agreement/consistency on WTC-based NVC (i.e., SctO2-aEEG coherence) exists between the different methods based on their total number of significant coherence pixels count across the entire wavelet scale. Furthermore, Fig. 6(d–f) panels demonstrate the consistency of numbers of significant coherence pixels determined by each pair of the three methods, by linearly regressing against a “line of identity”. Overall panels in Fig. 6 indicate the robustness of the WTC-based NVC based on the total number of significant coherence pixels across the wavelet scale, irrespective of the aEEG algorithm differences during the duration of the monitoring period.

## Discussion

We have established a new neurovascular wavelet bundle methodology which enables an unprecedented real time analysis of neurovascular coupling non-invasively at the bedside^4^. Key finding highlighted in this study is the robustness of NVC based on WTC analysis between aEEG signal and SctO2 in newborns with encephalopathy irrespective of processing algorithms variability in aEEGs.

In order to measure the neurovascular coupling with rigor, it is important that the dynamics of the signals are matched carefully. In general, these signals, typically emanating from electrical activity of neurons (EEG/aEEG) and optical absorption of light (NIRS) are associated with different time scales of function. An EEG signal undergoes fast changes due to desynchronization of numerous neurons, while a NIRS signal changes very slowly, due to the hemodynamic slow time scale^9^.

While all EEG to aEEG conversion methods follow some common initial pre-processing steps (see Fig. 1), such as asymmetrical band-pass filtering, rectification, and envelope detection, between-algorithm differences arise from practical parameters including gains applied on signal envelope, data segmentation, window durations, peak detection, and degree of smoothing technique. The amount of signal alteration depends on the degree of dissimilarity between methods. The above explains why different algorithms have resulted in significant differences in shape and amplitude margins of the aEEG output. This is consistent with the literature [4] and can impact clinical care as highlighted in the figure example. Our results agree with findings by Werther *et al*.^5^, which highlight the importance of careful selection of filters and band-pass range that can affect the tracing interpretation. The effect of the resulting differences in bandwidth shape and voltage on the full aEEG interpretation in both health and disease has not been studied to date.

These differences could be minimized by awareness of this particular issue and a careful selection of the algorithm settings^5^. The latter is not possible if these algorithms are undisclosed in commercial devices. These findings indicate that caution is needed prior to any clinical decision making for aEEG classification from automated tools and that vendors must be required to disclose all the details of their aEEG algorithm.

Despite the variabilities in the amplitude margins of the aEEG output, we observed consistent patterns in low frequency range of WTC-based NVC measured using the time-scale analysis between each of the three aEEG tracings and SctO2. The underlying basis of the observed robustness of the WTC-derived NVC to the different aEEG algorithms resides on the fact that the wavelet measures NVC via oscillations between two sets of time series via a time-frequency representation. When one wants to compute the interaction between two signals, it is important to define on which time scale the coupling is computed, and the signals have to be matched correspondingly. Some signals change very rapidly as EEG and are therefore mainly associated with high-frequency components, while others like NIRS change very slowly with the low frequency components.

Since the NIRS reflects the cardiovascular oscillations which develop at long-term scale, this results in down-sampling to a very low frequency around 0.21 Hz consistent with the SctO2 data acquisition and independent of the specific aEEG processing algorithm. When the WTC combines two time series, the high frequency components (vulnerable motion artifacts and other noises) are reduced, and low frequency aspects (related to physiological signals such as neurovascular coupling) are highlighted.

Note that the current findings are based on the NIRS SctO2 device used in this study which resulted in a sampling frequency of 0.21 Hz, so other NIRS devices could result in a different sampling rate but the same concepts of down sampling at low frequency would still apply. Also caution in the interpretation is indicated with absent or low NVC, as observations in Fig. 5 indicate that high NVC coupling is associated with a the least variation and/or less effect caused by different aEEG algorithms. We are currently testing the wavelet measurement of NVC in a larger cohort with asphyxia to ensure the validity of this physiological biomarker and its association with long-term clinical outcomes at two years.

## Conclusion

The study demonstrated that regardless of the differences in aEEG tracings the wavelet WTC-based NVC measures showed agreement when derived from three different aEEG algorithms. Findings suggest that NVC is a more robust parameter than automated aEEG patterns, supporting its validation and testing in a large group of newborns with hypoxic ischemic encephalopathy.
